# Pentachlorophenol and Cancer Risk: Focusing the Lens on Specific Chlorophenols and Contaminants

**DOI:** 10.1289/ehp.11081

**Published:** 2008-04-11

**Authors:** Glinda S. Cooper, Samantha Jones

**Affiliations:** National Center for Environmental Assessment, U.S. Environmental Protection Agency, Washington, DC, USA

**Keywords:** cancer, childhood leukemia, chlorophenols, dioxins, furans, multiple myeloma, non-Hodgkin lymphoma, pentachlorophenol, soft-tissue sarcoma

## Abstract

**Objective:**

Pentachlorophenol, a fungicide widely used as a wood preservative, was classified in 1999 by the International Agency for Research on Cancer as a possible human carcinogen. We reviewed currently available data to determine the extent to which recent studies assist in distinguishing the effect of pentachlorophenol from that of its contaminants (e.g., dioxins and other chlorophenols).

**Data sources and extraction:**

We performed a systematic review of published studies pertaining to cancer risk in relation to pentachlorophenol exposure, focusing on results pertaining specifically to all cancer sites and specific hematopoietic cancers, and data pertaining to risks associated with other types of chlorophenols, dioxins, or furans.

**Synthesis:**

The pentachlorophenol studies presented considerable evidence pertaining to hematopoietic cancers, with strong associations seen in multiple studies, in different locations, and using different designs. There is little evidence of an association between these cancers and chlorophenols that contain fewer than four chlorines. The extension of a large cohort study of sawmill workers, with follow-up to 1995, provided information about risks of relatively rare cancers (e.g., non-Hodgkin lymphoma, multiple myeloma), using a validated exposure assessment procedure that distinguishes between exposures to pentachlorophenol and tetrachlorophenol. In contrast with dioxin, pentachlorophenol exposure has not been associated with total cancer incidence or mortality.

**Conclusions:**

The updated cohort study focusing on pentachlorophenol provides increased statistical power and precision, and demonstrates associations between hematopoietic cancer and pentachlorophenol exposure not observed in earlier evaluations of this cohort. Contaminant confounding is an unlikely explanation for the risks seen with pentachlorophenol exposure.

Pentachlorophenol (CAS Registry no. 87-86-5; also referred to as penta, pentachlorofenol, 2,3,4,5,6-pentachlorophenol, and chlorophen) is a chlorinated aromatic compound that has been used extensively as a fungicide. Impurities comprise approximately 10% of technical or commercial grade pentachlorophenol and consist of several congeners of the chlorophenols, primarily the higher chlorinated congeners of dibenzo-*p*-dioxins and dibenzofurans [National Toxicology Program (NTP) 1989; Schwetz et al. 1974]. Pentachlorophenol was first registered as a wood preservative in the United States in 1936 (Ahlborg and Thunberg 1980) and has also been used in ropes, paints, adhesives, canvas, insulation, and brick walls [Agency for Toxic Substances and Disease Registry (ATSDR) 2001; Proudfoot 2003]. Use by the general public was restricted in 1984, and pentachlorophenol application was limited to industrial areas (e.g., utility poles, railroad crossties, fence posts). The 95th percentile of urinary pentachlorophenol concentration was approximately 1.0–2.0 μg/L in the 1999–2002 National Health and Nutrition Examination Survey III [Centers for Disease Control and Prevention (CDC) 2005]. The comparable figure from a population-based study in Germany in 1998 was 3 μg/L (Schulz 2007); in that study, levels had decreased since 1990.

Case reports (Bishop and Jones 1981; Greene et al. 1978) and case–control studies of hematopoietic cancers published in the 1980s and 1990s (Hardell et al. 1981, 1994, 1995; Kogevinas et al. 1995; Pearce et al. 1986b; Smith and Christophers 1992; Smith et al. 1984; Woods et al. 1987) raised concerns about the risk of non-Hodgkin lymphoma and soft-tissue sarcoma in relation to pentachlorophenol exposure. Other studies examined the carcinogenic effect of pentachlorophenol in experimental animals. Some of the most extensive animal studies were conducted by the NTP in B6C3F_1_ mice (NTP 1989) and F344 rats (NTP 1999). The type of cancer seen among the exposed animals included hepatocellular adenomas and carcinomas in male and female mice (a relatively common cancer among these animals, particularly in the males); rarer types of cancer including adrenal medullary pheochromocytomas in male and female mice, hemangiosarcomas in female mice, and malignant mesothelioma and nasal squamous cell carcinoma in male rats.

Evaluation of available literature conducted in the 1990s by the International Agency for Research on Cancer (IARC) and the U.S. Environmental Protection Agency (EPA) classified pentachlorophenol as a “possible human carcinogen” based on sufficient information in animal assays but limited data in humans (IARC 1999; U.S. EPA 1991). The U.S. EPA assessment, completed in 1991, was conducted before the publication of most of the case–control and cohort studies focusing on pentachlorophenol exposure. IARC, in 1999, noted that the most consistent findings among the studies available at that time were for soft-tissue sarcoma and non-Hodgkin lymphoma. One concern noted about the data was the potential effect of recall bias in the case–control studies, although this was thought to be an unlikely explanation for all of the observed associations. Another concern noted in the IARC summary (IARC 1999) was the perceived inability to distinguish a possible confounding effect of the contaminants, particularly polychlorinated dibenzo-*p*-dioxins (PCDDs), within chlorophenols.

We reviewed the currently available epidemiologic studies of cancer risk in relation to pentachlorophenol exposure to explore the availability of relevant data pertaining to distinguishing the effect of pentachlorophenol from that of other chlorophenols and from dioxins. Additionally, we reviewed studies to determine the extent to which more recent studies address the limitations of the previous data.

## Methods

We searched the MEDLINE database (National Library of Medicine, Bethesda, MD) for studies related to pentachlorophenols and cancer risk, using “pentachlorophenol” and “chlorophenol” as search terms. We also reviewed references within relevant reports.

We included studies in this review if information was presented pertaining specifically to pentachlorophenol exposure. In the absence of either a quantitative or qualitative pentachlorophenol measure, we also accepted studies that included an assessment of chlorophenol exposure and additional information on specific jobs that would have likely used pentachlorophenol rather than other chlorophenols. We excluded two cohort studies from workplaces with exposures to other carcinogens linked to hematopoietic cancers because data were not presented that allowed estimation of effects specifically for pentachlorophenol. These excluded studies evaluated formaldehyde-exposed workers in a plywood mill (Robinson et al. 1987) and a Swedish tannery cohort with a variety of exposures, including formaldehyde and azo and benzidine dyes (Mikoczy and Hagmar 2005). Pentachlorophenol represented < 10% of the chlorophenol exposure in a cohort study of sawmill workers in Finland (Jäppinen et al. 1989) and in a study of cancer incidence in the area surrounding a mill (Lampi et al. 1992), and these studies were also excluded. We excluded studies that presented data only for a combined exposure such as chlorophenols, or chlorophenols and phenoxy herbicides (Garabedian et al. 1999; Hooiveld et al. 1998; Hoppin et al. 1998; Kogevinas et al. 1997; Ott et al. 1987; van Balen et al. 2006; Wingren et al. 1990). The other studies we identified but did not include were two small surveys (< 200 participants) of exposed workers that contained very limited information pertaining to cancer mortality (Cheng et al. 1993; Gilbert et al. 1990), as well as studies of nasopharyngeal cancer (Mirabelli et al. 2000; Zhu et al. 2002).

## Results

### Case–control studies of hematopoietic cancers

The case–control studies of pentachlorophenol and specific hematopoietic cancer were conducted between 1970 and 1995 (Tables 1 and 2). Two case–control studies of non-Hodgkin lymphoma (Pearce et al. 1986b; Woods et al. 1987), one study that combined Hodgkin and non-Hodgkin lymphoma (Smith and Christophers 1992), two studies of soft-tissue sarcoma (Smith et al. 1984; Woods et al. 1987), and one of multiple myeloma (Pearce et al. 1986a) assessed occupational exposure to chlorophenols with limited data specifically relating to potential exposure to jobs or activities with likely exposure to pentachlorophenol. These studies reported no or weak associations [odds ratios (ORs) < 1.5] with chlorophenols, but showed somewhat stronger risks with some specific jobs involving wood preservation or fencing work (jobs generally associated with pentachlorophenol).

More detailed assessment of pentachlorophenol exposure was presented within the population-based case–control studies of non-Hodgkin lymphoma (Hardell et al. 1994) (Table 1) and soft-tissue sarcoma (Hardell et al. 1995) conducted in Sweden. The 1995 meta-analysis of soft-tissue sarcoma (Hardell et al. 1995) was based on four previously published studies (Erikkson et al. 1981, 1990; Hardell and Eriksson 1988; Hardell and Sandrstrom 1979) that had presented data pertaining to chlorophenols (Table 2). The reanalysis used the original study data to generate estimates specifically for pentachlorophenol. In these studies from Sweden, cases were identified through hospital records or a cancer registry, and controls were identified through a population registry (for matching to living cases) and the national death registry (for matching to deceased cases). With the exception of one of these studies (Hardell and Eriksson 1988), the matching process resulted in an equal proportion of deceased cases and controls. A self-administered questionnaire with follow-up telephone interview, if needed, was used to obtain detailed information pertaining to work history, including information on specific jobs and exposures. Next-of-kin proxy respondents were used for deceased cases and controls. This information was used to create an exposure measure for specific chemicals, including chlorophenols and pentachlorophenols. Exposures in the 5 years immediately preceding diagnosis (or a corresponding reference year for controls) were excluded to account for a minimum latency period. High exposure was defined as 1 week or more continuously or at least 1 month total. A strong association [OR = 8.8; 95% confidence interval (CI), 3.4–24] was observed between high exposure to pentachlorophenol and risk of non-Hodgkin lymphoma (Hardell et al. 1994) and in the meta-analysis of four soft-tissue sarcoma studies (OR = 2.8; 95% CI, 1.5–5.4 for high pentachlorophenol exposure) (Hardell et al. 1995).

A nested case–control study within a large, international cohort of workers exposed to phenoxy herbicides or chlorophenols (Kogevinas et al. 1995) also provided a more detailed assessment of pentachlorophenol and related exposures. Job records and company records pertaining to chemicals used during specific processes were used by three industrial hygienists to evaluate exposure to 21 specific chemicals [phenoxy herbicides, chlorophenols, PCDDs and polychlorinated dibenzofurans (PCDFs), process chemicals, and raw materials]. The estimated associations with non-Hodgkin lymphoma in that study are relatively imprecise, given the small size (*n* = 32 cases and 158 controls), but there is evidence of an association with any pentachlorophenol exposure (OR = 2.75; 95% CI, 0.45–17.0) and specifically with the high cumulative exposure category (OR = 4.19; 95% CI, 0.59–29.6). In contrast with the elevated point estimates seen with pentachlorophenol, there was no evidence of an association with the chlorophenols containing fewer than four chlorines (ORs = 0.65, 0.80, and 1.03 for 2,4,5-trichlorophenol, 2,4,6-trichlorophenol, and 2,4-dichlorophenol, respectively). No estimate was provided for 2,3,4,6-tetrachlorophenol because of lack of model convergence (1 exposed case and 7 exposed controls). The associations seen with dioxins appeared somewhat weaker than those seen with pentachlorophenol [ORs = 1.84 for any dioxin or furan, 1.93 for 2,3,7,8-tetrachlorodibenzo-*p*-dioxin (TCDD), and 2.75 for pentachlorophenol], although the small sample size makes precise comparisons difficult. There were only 12 cases of soft-tissue sarcoma identified within the cohort, and none of these cases or the 44 matched controls had been exposed to pentachlorophenol.

### Cohort studies in pentachlorophenol-exposed workers

Cohort studies of workers exposed to pentachlorophenol in sawmills and in a manufacturing plant have been conducted (Table 3). Ramlow et al. (1996) examined the mortality risk in a cohort of 770 male workers at Dow Chemical Company, Michigan Division. This plant manufactured pentachlorophenol from the late 1930s to 1980. Exposure to dioxins, primarily hexa-, hepta-, and octa-CDDs and CDFs also occurred within this cohort (Collins et al. 2007; Ott et al. 1993). Potential for exposure to pentachlorophenol was assessed by evaluating available industrial hygiene data, including some quantitative environmental and personal breathing zone measurements of pentachlorophenol in conjunction with detailed employment records. Each job was assigned an estimated exposure intensity score and the estimated cumulative exposure index was calculated for each subject based on the product of job duration and intensity, summed across all jobs. Collins et al. (2007) and Ott et al. (1993) used a similar process to estimate cumulative exposure to TCDD and the hexa-CDD to octa-CDD ratio. Mortality risk for all causes of cancer was not elevated, and there were no deaths due to soft-tissue sarcoma or Hodgkin disease. Increased standardized mortality ratios (SMRs) were seen for some forms of lymphopoietic cancers (Table 3), particularly in the high-exposure group (defined as cumulative exposure ≥ 1). Similar results were seen with a 15-year latency period. There was some indication of an increased risk of lymphopoietic cancer with the dioxin measures, but these effects were somewhat weaker than those seen with pentachlorophenol. (For all lymphopoietic cancers, trend *p*-value = 0.34 for TCDD, 0.53 for the hexa-CDD to octa-CDD ratio, and 0.08 for pentachlorophenol.)

Another larger cohort study involved > 26,000 male sawmill workers from 14 mills in British Columbia, Canada (Hertzman et al. 1997). This study was recently updated by Demers et al. (2006) in an analysis that included specific measures of pentachlorophenol and tetrachlorophenol exposures and with an extension of the follow-up period from 1989 to 1995 (Table 3). Demers et al. (2006) included 26,487 men who had worked at least 1 year (or 260 days total) between 1950 and 1995, with 2,658 workers from the mills that did not use the fungicides included in the unexposed group in the exposure–response analyses. The authors used record linkage through the provincial and national death files and cancer incidence registries to assess mortality (from first employment through 1995) and cancer incidence (from 1969, when the provincial cancer registry began, through 1995). Plant records were available to determine work histories for study cohort members, including duration of work within different job titles. Representative exposures were determined for three or four time periods for each mill. Demers et al. (2006) developed a retrospective exposure assessment based on interviews with senior workers (≥ 5 years of experience) at each mill (9–20 workers for each time period; mean, 15 years of experience). For current exposures, the process was compared with urinary measurements, resulting in correlation coefficients of 0.76 and 0.72 in two different sampling periods (Hertzman et al. 1988). The validity of this method was also demonstrated in comparison with a method based on industrial hygienist assessment (Teschke et al. 1989, 1996). A cumulative dermal chlorophenol exposure score was calculated for each worker by summing, across all jobs, the product of the job-title–specific exposure score and the length of employment in that job. Records from each mill were used to determine the specific chlorophenol content of the fungicides used at specific time periods. In general, tetrachlorophenol was used increasingly in place of pentachlorophenol after 1965. This information was used to develop pentachlorophenol-and tetrachlorophenol-specific exposure scores. The correlation between the estimated pentachlorophenol and tetrachlorophenol exposures was 0.45 (Demers et al. 2006).

Hertzman et al. (1997) reported only a weak association between the total chlorophenol measure and non-Hodgkin lymphoma risk. Based on 65 cases, the standardized incidence ratios (SIRs) across five levels of cumulative exposure to chlorophenates were 0.68, 0.59, 1.04, 10.2, and 1.30. No association was seen with risk of multiple myeloma (SIRs 1.18, 0.93, 0.66, 0.19, and 1.11, across the exposure levels, based on 19 cases). However, in the more recent analysis of pentachlorophenol exposure, Demers et al. (2006) observed exposure effects for non-Hodgkin lymphoma and multiple myeloma. Table 4 presents the results from Demers et al.’s incidence data. The authors also presented a similar set of results from the analysis of the mortality data. Liver cancer was also associated with pentachlorophenol exposure. Analyses using a 10-year (data not shown) or 20-year (Table 4) latency period showed similar or stronger associations with respect to pentachlorophenol exposure and risk of non-Hodgkin lymphoma (trend *p*-value = 0.02) and multiple myeloma (trend *p*-value = 0.03), but not liver cancer (trend *p*-value = 0.38). Similar results were also seen in the analyses using a 10-year latency period presented by Demers et al. (2006). The risk of non-Hodgkin lymphoma or multiple myeloma in relation to tetrachlorophenol (Table 4) was somewhat smaller than for pentachlorophenol, particularly in the analysis including a 20-year latency period. The number of incident cases of soft-tissue sarcoma was small (*n* = 23), and lower risks of this cancer were seen in the higher exposure groups for pentachlorophenol and for tetrachlorophenol.

### Studies of childhood cancers

A recent cohort study in Canada (Heacock et al. 2000) and a case–control study in Taiwan (Ali et al. 2004) examined childhood cancer risk in relation to parental occupational exposure to pentachlorophenol. The cohort study (Heacock et al. 2000) was based in the offspring of the male workers in the British Columbia sawmill workers cohort studied by Demers et al. (2006). In this analysis of childhood cancers, however, the exposure metric was based on total chlorophenol exposure, rather than on separate estimates for pentachlorophenol and tetrachlorophenol. Marriage and birth records were linked to identify 19,675 children born to these fathers between 1952 and 1988. Forty incident childhood cancers (including 11 leukemias, 9 brain cancers, and 4 lymphomas) were identified among these children (with follow-up through 19 years of age) through the linking of birth records to the provincial cancer registry. The incidence rates were similar to those expected based on sex-, age-, and calendar year–standardized rates, with a standardized incidence rate of 1.0 (95% CI, 0.7–1.4) for all cancers, 1.0 (95% CI, 0.5–1.8) for leukemia, and 1.3 (95% CI, 0.6–2.5) for brain cancer.

Ali et al. (2004) conducted a population-based case–control study of leukemia (*n* = 103 cases) and brain cancer (*n* = 74 cases) in patients diagnosed before 30 years of age in Taiwan. Occupational history for jobs held > 6 months since 16 years of age was obtained using a structured interview with each of the parents and any patient (or control) who was at least 16 years of age. The analysis focused on specific periods of exposure (e.g., preconception defined as any job ending > 1 year before the child’s birth; perinatal, any job held between 1 year before the child’s birth and the child’s birth). Adjusting for smoking history (for the participant and the parents) and exposure to medical radiation, Ali et al. (2004) observed strong but statistically imprecise associations between paternal work as a wood treater and risk of leukemia: for any exposure period, 5 exposed cases and 2 exposed controls (OR = 16.0; 95% CI, 1.8–145.4); for the preconception period, 4 exposed cases and 1 exposed control (OR = 12.2; 95% CI, 1.4–109.2); for the perinatal period, 4 exposed cases and 1 exposed control (OR = 13.0; 95% CI, 1.4–125.5). No other information is available pertaining to the specific material used by these workers (Christiani D, personal communication).

## Discussion

The available epidemiologic studies present evidence pertaining to cancer risk and occupational exposure to pentachlorophenol, particularly with respect to hematopoietic cancers. The literature base is quite broad, with multiple studies, in different locations, and using different designs. The more recent studies have allowed more focused analysis, specifically of risks associated with pentachlorophenol exposure. In addition, recent studies have demonstrated a relatively strong ability of pentachlorophenol (compared with other organochlorine pesticides tested) with respect to inhibition of the cytotoxic activity of natural killer cells (Reed et al. 2004; Taylor et al. 2005), an effect of particular relevance given the role of natural killer cells in tumor surveillance (Smyth et al. 2002).

### Synthesis of studies of pentachlorophenol and hematopoietic cancers

The strongest of the available studies, in terms of design, is the large sawmill cohort study conducted in British Columbia, Canada, and recently updated by Demers et al. (2006). This study used a population-based cancer registry, which allowed for the analysis of cancer incidence. The exposure assessment procedure was developed specifically to address the exposure situations and setting of the work-site, and the refined exposure metric provides an ability to distinguish effects of pentachlorophenol and tetrachlorophenol. Recall bias is not an issue given the design of the study, in which the exposure assessment is derived independently of the cancer classification and is based on company records rather than self-reported work histories. Common behaviors, such as smoking and use of alcohol, have not been associated with the types of cancers associated with pentachlorophenol exposure in this study (non-Hodgkin lymphoma, multiple myeloma), so it is difficult to conceive of a way in which the observed associations could be explained by confounding by those factors. In addition, the use of an internal comparison group for the analyses reduces the likelihood of other potential confounders affecting the results. No information is provided about the effect of adjustment for tetrachlorophenol exposure on the pentachlorophenol results. However, because the correlation between the two measures is relatively low (*r* = 0.45), and for many of the cancers of interest the pentachlorophenol associations are stronger than those seen with tetrachlorophenol, it is unlikely that this adjustment would attenuate the observed associations with pentachlorophenol.

However, even with a cohort study of this size, there is limited statistical power to estimate precise associations with relatively rare cancers. Thus, the data from this cohort study are complemented by the data from case–control studies of specific lymphopoietic cancers. Strong associations were seen in a series of case–control studies in Sweden between high (defined as > 1 week continuously or 1 month total) exposure to pentachlorophenol and non-Hodgkin lymphoma (Hardell et al. 1994) and soft-tissue sarcoma (Hardell et al. 1995), with ORs of 8.4 and 2.8, respectively. In a small nested case–control study of non-Hodgkin lymphoma (32 cases, 158 controls), elevated point estimates (with wide CIs) were seen with pentachlorophenol and high pentachlorophenol exposure measures, but there was no evidence of an association (i.e., ORs less than or approximately equal to 1.0) with the di- and trichlorophenols (Kogevinas et al. 1995). Several limitations of individual studies can be noted, including the imprecision of the estimates from the very smallest study and the difficulty in obtaining accurate work histories from proxy respondent in studies with a rapidly fatal disease. Evaluating the collection of studies, however, allows for a more considered analysis of the potential effect of these limitations. For example, concern about the effect of differential misclassification of exposure would be reduced if, as is the case in this set of studies, the cohort and nested case–control studies, in which exposure assessment did not depend on interview data, generally support the data from case–control studies relying on self-reported job histories.

One limitation to the observations from the sawmill cohort study that should be noted is the temporal difference in pentachlorophenol and tetrachlorophenol exposures, with tetrachlorophenol replacing pentachlorophenol beginning around 1965. Thus, a longer follow-up period may be needed to fully capture the effect of tetrachlorophenol if a long latency period (≥ 20 years) is assumed.

The classifications used for the various subtypes of lymphomas, leukemias, and sarcomas can be confusing and may not be applied similarly in different studies, particularly when conducted over different time periods or in different locations by different investigators. Any differences in disease definitions should not produce a biased result within a study, given that the disease classification methods in the available studies were independent of the exposure classification system. It is not clear if potential inconsistencies in disease classifications contributed to the difference seen with respect to soft-tissue sarcoma and pentachlorophenol exposure in Sweden compared with the sawmill cohort in Canada, or if this difference reflects a difference in population susceptibility, other factors, or chance.

Childhood leukemia and brain cancer risks were not increased among children of the sawmill workers in an analysis using the chlorophenol, rather than the specific pentachlorophenol and tetrachlorophenol, measures (Heacock et al. 2000). In light of the data from the case–control study by Ali et al. (2004), however, additional studies with focused exposure assessments for paternal or maternal pentachlorophenol exposure may be needed to determine if prenatal or early-life exposures to pentachlorophenol influences the risk of childhood cancers.

### Distinguishing effects of specific chlorophenols and contaminant

Contamination of pentachlorophenol with dioxins and related by-products occurs as part of the production process, and the possible confounding effects of these contaminants (particularly PCDDs) is an important consideration in the evaluation of the studies of manufacturing workers (Kogevinas et al. 1995; Ramlow et al. 1996). In a recent follow-up study, Collins et al. (2007) reported an increased mean lipid-adjusted serum level of TCDD in pentachlorophenol production workers from the Dow Chemical Company Michigan plant (8.0 pg/g lipid) compared with community referents (3.3 pg/g lipid) or other worker referents (6.5 pg/g lipid) in the plant. These measurements were taken > 20 years after production work at the plant had ended. The epidemiologic studies of TCDD have consistently demonstrated associations with total cancer mortality, with a combined SMR of 1.4 (95% CI, 1.1–1.7) seen in a recent analysis of four cohorts (Steenland et al. 2004). In contrast, the SMR for all cancer mortality in the pentachlorophenol manufacturing workers was 0.95 (Ramlow et al. 1996). The dissimilarity between the observed patterns in cancer risk for pentachlorophenol and TCDD makes it unlikely that confounding by this compound is a reasonable explanation for the specific cancer risks seen with pentachlorophenol exposure.

Other forms of dioxins (e.g., specific hexa-, hepta-, and octa-CDDs) were substantially elevated in the pentachlorophenol production workers in the Collins et al. (2007) follow-up study, with levels for some of the congeners two to four times higher compared with community referents, worker referents, and trichlorophenol production workers in the plant. For example, mean 1,2,3,6,7,8-hexa-chlorodibenzo-*p*-dioxin levels were 150.6 pg/g lipid in the pentachlorophenol production workers compared with 68–78 pg/g lipid in the other groups; comparable values for octachlorodibenzo-*p*-dioxin were 2,594 pg/g lipid in the pentachlorophenol and 439–616 pg/g lipid in the other groups. Few studies have examined risks associated with these forms of dioxin, but there was no association between plasma levels of 1,2,3,6,7,8-hexaCDD, 1,2,3,4,5,6,8-heptaCDD, or octaCDD levels and lymphoma risk in a recent case–control study of 100 non-Hodgkin lymphoma cases and 100 controls (De Roos et al. 2005).

TCDD has not been detected in technical-grade or commercial-grade pentachlorophenol (NTP 1989; Schwetz et al. 1974). In studies of pentachlorophenol users (e.g., in sawmill or other lumber operations), concern about contaminant confounding should thus focus on the major pentachlorophenol contaminants, hexachlorinated dibenzodioxins, dibenzofurans, and chlorophenols (particularly the primary contaminant, tetrachlorophenol). As described previously, two studies included measures of pentachlorophenol and tetrachlorophenol (Demers et al. 2006; Kogevinas et al. 1995), and these studies suggested stronger associations seen between multiple myeloma and non-Hodgkin lymphoma and pentachlorophenol than between these cancers and tetrachlorophenol.

Animal evidence also suggests that the contaminants are an unlikely explanation for the carcinogenicity associated with pentachlorophenol. Male rats exposed to a pure formulation of pentachlorophenol (i.e., analytical grade pentachlorophenol, which does not contain chlorophenols, dioxin, or furan contaminants) exhibited nasal squamous cell carcinomas and mesotheliomas (NTP 1999). The technical-grade pentachlorophenol formulation used in the NTP bioassay (NTP 1989) comprises approximately 90% pentachlorophenol, 4% tetrachlorophenol, and 6% chlorohydroxydiphenyl ethers. The commercial-grade (EC-7) formulation is composed of approximately 91% pentachlorophenol and 9% tetrachlorophenol. Both formulations of pentachlorophenol induced liver tumors in male and female mice (NTP 1989). In the mouse studies, the tumor response was similar, with only slightly stronger responses with the technical grade compared with commercial grade. Tetrachlorophenol likely plays a minimal role in the carcinogenicity of pentachlorophenol, as the difference in potencies seen in the bioassays is inversely related to the relative amounts of tetrachlorophenol in each formulation (i.e., 4% in technical-grade pentachlorophenol vs. 9% in commercial-grade pentachlorophenol).

Another group of contaminants reported is hexaCDDs, which comprise 0.001% of technical-grade pentachlorophenol and 0.00002% of commercial-grade pentachlorophenol (NTP 1989). The tumor responses observed with the two formulations of pentachlorophenol were similar, although technical-grade pentachlorophenol showed a slightly greater potency relative to commercial-grade pentachlorophenol. It is unlikely that hexaCDD is responsible for the carcinogenicity observed in mice exposed to pentachlorophenol, considering there was a 50-fold difference in the amount of this hexaCDD congener in the technical-grade and EC-7 pentachlorophenol formulations.

## Conclusions

Recent studies have improved our ability to determine the specific role of pentachlorophenol compared with other chlorophenols and contaminants in evaluating cancer risk. These studies include the updated analysis of the large cohort of sawmill workers (Demers et al. 2006), serologic measurements of contaminant profiles in manufacturing workers (Collins et al. 2007), and a more comprehensive understanding of the risks associated or not associated with specific forms of dioxins (De Roos et al. 2005; Steenland et al. 2004). The updated cohort study provides improved precision for associations with cancer incidence and mortality, with separate exposure estimates for tetrachlorophenol and pentachlorophenol in a work setting without concurrent exposure to TCDD. Human and animal data support the idea that the associations seen with specific cancers and pentachlorophenol cannot be explained by contamination with tetrachlorophenol. These new data provide a more focused framework upon which the earlier case–control studies can be evaluated and also provide support for the idea that these earlier results cannot be attributed to other substances or to recall bias. Additional research pertaining to pentachlorophenol and cancer risk should focus on the potential role in childhood cancers and questions pertaining to factors affecting the development of specific forms of hematopoietic cancers. The currently available data do indicate, however, that specific hematopoietic cancer risks are observed with pentachlorophenol exposure and that these risks are not likely to be due to dioxins or other chlorophenol contaminants.

## Figures and Tables

**Table 1:** Case–control studies of non-Hodgkin lymphoma, soft-tissue sarcoma, and multiple myeloma risk and in relation to pentachlorophenol exposure.

Disease	Reference, location, demographic data	Cases and controls [no. (source)], source of exposure data	Results
Non-Hodgkin lymphoma			
	Pearce et al. 1986b New Zealand Men, age < 70 years	83 cases (cancer registry) 168 cancer controls and 228 population controls Structured interview	Chlorophenols: OR = 1.3 (95% CI, 0.6–2.7) Fencing work: OR = 2.0 (95% CI, 1.3–3.01)
	Woods et al. 1987 USA (Washington State) Males, age 20–79 years	576 cases (cancer registry) 694 population controls Structured interview	Chlorophenols: OR = 0.99 (95% CI, 0.8–1.2) Increased risk (OR > 1.5) for wood preservers and chlorophenols manufacturers but not for lumber grader (OR = 0.94)
	Hardell et al. 1994 Sweden Males, age 25–85 years	105 cases (hospital records); 355 population controls Self-administered questionnaire with follow-up telephone interview if needed	High (> 1 week continuously or 1 month total) exposure to pentachlorophenols: OR = 8.8 (95% CI, 3.4–24)
	Kogevinas et al. 1995 Europe Primarily male (31 of 32)	32 cases (death certificates for all countries; cancer registries for seven countries) 158 controls (nested case–control study within a cohort study of exposed workers)^a^ Company records and industrial hygienist review; cumulative exposure scores for various chlorophenols and dioxins	Pentachlorophenols: OR = 2.75 (95% CI, 0.45–17.0) High pentachlorophenols: OR = 4.19 (95% CI, 0.59–29.6)
Non-Hodgkin lymphoma and Hodgkin lymphoma			
	Smith and Christophers 1992 Australia Males, age ≥ 30 years	52 cases (cancer registry), 52 cancer controls and 52 population controls Deceased cases and controls excluded Structured interview	Chlorophenols: OR = 1.4 (95% CI, 0.3–6.1) 4 cases and 4 controls (1 population and 3 cancer controls) had definite pentachlorophenol exposure.
Soft-tissue sarcoma			
	Smith et al. 1984 New Zealand Males, age 20–80 years	82 cases (cancer registry), 92 cancer controls Structured interview	Chlorophenols: OR = 1.5 (95% CI, 0.5–4.5) Variable results (ORs from 0.7 to 1.9) for fencing and sawmill/timber merchant jobs
	Woods et al. 1987 USA (Washington State) Males, age 20–79 years	128 cases (cancer registry), 694 population controls Structured interview	Chlorophenols: OR = 0.99 (95% CI, 0.7–1.5) Lumber grader: OR = 2.7 (95% CI, 1.1–6.4) Variable results (ORs from 0.79 to 4.8) for other high-, medium-, and low-exposure jobs
	Smith and Christophers 1992 Australia Males, age ≥ 30 years	30 cases (cancer registry), 30 cancer controls and 52 population controls Deceased cases and controls excluded Structured interview	Chlorophenols ≥ 1 day: no cases with this exposure; no cases and 2 controls (1 population and 1 cancer control) had definite pentachlorophenol exposure
	Hardell et al. 1995 Meta-analysis of four studies^b^ Sweden Males, ages 25–80 years	434 cases (hospital records; cancer registry), 948 population controls Self-administered questionnaire with follow-up telephone interview if needed	High (more than 1 week continuously or 1 month total) exposure to pentachlorophenols: OR = 2.8 (95% CI, 1.5–5.4)
	Kogevinas et al. 1995 Europe Males	12 cases (death certificates for all countries; cancer registries for 7 countries), 44 controls (nested case–control study within cohort study of exposed workers^a^) Company records and industrial hygienist review, cumulative exposure scores for various chlorophenols and dioxins	Pentachlorophenols: no exposed cases or controls
Multiple myeloma			
	Pearce et al. 1986a New Zealand Males, age < 70 years	76 cases and 315 cancer controls	No association (OR = 1.1) with chlorophenol exposure, work in a sawmill or timber merchant, or treating fence posts Stronger associations with fencing work (OR = 1.6; 95% CI, 0.9–2.7) and jobs at a sawmill or timber merchant that involved potential exposure to chlorophenols (OR = 1.4; 95% CI, 0.5–3.9)

a Twenty-four cohorts from 11 countries; total n = 21,183 workers, exposures to phenoxy herbicides or chlorophenols.

b Hardell and Sandstrom (1979), Eriksson et al. (1981), Hardell and Eriksson (1988), and Eriksson et al. (1990).

**Table 2:** Case–control studies of chlorophenol and soft-tissue sarcoma risk included in Hardell et al. meta-analysis (1995).

Reference	Swedish region, case accrual, sex and age	Cases [no. (percent deceased)] and controls (no.)^a^	Results^b^
Hardell and Sandstrom 1979	Umeå (northern Sweden)	52 cases (60% deceased)	Any chlorophenols
	1970–1977, hospital records Males, ages 26–80 years	208 controls	OR = 6.6 (*p* < 0.001)
Eriksson et al. 1981	Five counties (southern Sweden)	110 cases (35% deceased)	Any chlorophenols
	1974–1978, cancer registry Sex and age not specified	220 controls	OR = 3.3 (95% CI, 1.3–8.1)
Hardell and Eriksson 1988	Three counties (northern Sweden)	54 (67% deceased),	Any chlorophenols, no association
	1978–1983, cancer registry Males, ages 25–80 years	311 controls (33% deceased)	
Eriksson et al. 1990	Upsala (central Sweden)	218 (64% deceased)	Low chlorophenols
	1978–1986, cancer registry Males, ages 25–80 years	212 controls	OR = 0.89 (95% CI, 0.40–2.0) High chlorophenols OR = 5.24 (95% CI, 1.69–16.3)

a The matching design used in all of the studies except Hardell and Eriksson (1988) resulted in an equal proportion of deceased cases and controls within each study.

b The publications presented data pertaining to chlorophenols, but reanalysis with the meta-analysis (Hardell et al. 1995) used original study data to generate estimates specifically for pentachlorophenol.

**Table 3:** Summary of cohort studies of cancer risk in pentachlorophenol exposed workers.

Cohort, location	Reference	Total no., duration of work, follow-up, inclusion criteria	Exposure assessment, outcome assessment	Results^a^
Dow manufacturing plant, USA (Michigan)				
	Ramlow et al. 1996	*n* = 770 men Mean duration: not reported Mean follow-up: 26.1 years Worked some time between 1937 and 1980 in a relevant department	Work history (job records) and industrial hygiene assessment; developed exposure intensity and cumulative exposure scores for pentachlorophenol and for dioxins^b^ Death certificate (underlying cause)	All cancers: SMR = 0.95 Elevated risk of lymphatic cancer mortality, particularly at higher intensity exposures Any pentachlorophenol exposure All lymphopoietic cancers (seven cases): SMR = 1.4 (95% CI, 0.56–2.88) Other and unspecified lymphopoietic cancers (five cases):^c^ SMR = 2.0 (95% CI, 0.65–4.7) High exposure (cumulative exposure ≥ 1) All lymphopoietic cancers: SMR = 1.91 (95% CI, 0.86–4.24) Trend *p*-value = 0.08 Other and unspecified lymphopoietic cancers: SMR = 2.58 (95% CI, 0.98–6.8) Trend *p*-value = 0.19
Sawmill workers, Canada (British Columbia)				
	Hertzman et al. 1997	*n* = 23,829 men (1,498 incident cancer cases; 105 cancer deaths) Mean duration: 9.8 years Mean follow-up: 24.5 years Worked at least 1 year (or 260 days total) between 1950 and 1985; follow-up through 1989	Work history (job records) and industrial hygiene assessment; developed cumulative exposure scores for total chlorophenols Death certificate (underlying cause) and cancer registry (incidence)	Weak or no risk of non-Hodgkin lymphoma and multiple myeloma
	Demers et al. 2006	Same as Hertzman et al. 1997 except follow-up through 1995 2,571 incident cancer cases 1,495 cancer deaths	Same as Hertzman et al. 1997 except that cumulative exposure scores were developed for pentachlorophenol and tetrachlorophenol	All cancers: SMR = 1.00, SIR = 0.99 Elevated risk of non-Hodgkin lymphoma and multiple myeloma incidence and mortality; evidence of exposure–effect response; weaker or no risk seen with tetrachlorophenol (Table 4)

SIR, standardized incidence ratio.

a Elevated indicates an SMR of ≥ 1.5.

b TCDD and the hexa-CDD to octa-CDD ratio.

c Two of these cases were multiple myeloma and three cases would now be classified as non-Hodgkin lymphoma.

**Table 4:** Cancer incidence risk in relation to estimated pentachlorophenol and tetrachlorophenol exposure in sawmill workers, British Columbia, Canada.^a^

		Pentachlorophenol exposure						Tetrachlorophenol exposure					
		No latency			20-year latency			No latency			20-year latency		
Cancer	Exposure-years	Obs	RR	95% CI	Obs	RR	95% CI	Obs	RR	95% CI	Obs	RR	95% CI
Non-Hodgkin lymphoma	< 1	38	1.0	(Referent)	46	1.0	(Referent)	50	1.0	(Referent)	78	1.0	(Referent)
	1–2	13	1.3	0.70–2.5	13	1.8	0.59–3.5	11	0.90	0.47–1.8	8	1.7	0.78–3.5
	2–5	24	1.9	1.1–3.3	21	2.1	1.1–3.7	20	1.3	0.80–2.3	5	1.3	0.49–3.3
	≥ 5	17	1.7	0.91–3.2	12	2.0	0.97–4.1	11	1.5	0.79–3.0	1	1.5	0.20–11.1
	(Trend^b^)			(0.06)			(0.02)			(0.14)			(0.32)
Multiple myeloma	< 1	6	1.0	(Referent)	8	1.0	(Referent)	15	1.0	(Referent)	19	1.0	(Referent)
	1–2	4	2.1	0.57–7.6	3	1.7	0.43–7.0	1	0.27	0.04–2.0	3	1.7	0.48–5.9
	2–5	4	1.3	0.34–5.0	6	2.1	0.62–7.0	5	1.1	0.38–2.9	3	1.8	0.48–6.7
	≥ 5	11	4.2	1.4–12.9	8	3.8	1.2–12.3	4	1.8	0.58–5.6	0		
	(Trend)			(0.02)			(0.03)			(0.48)			(0.30)
Soft-tissue sarcoma	< 1	18	1.0	(Referent)	20	1.0	(Referent)	16	1.0	(Referent)	23	1.0	(Referent)
	1–2	3	0.64	0.18–2.2	1	0.34	0.04–2.6	3	0.77	0.23–2.66	0		
	2–5	2	0.18	0.04–0.85	2	0.33	0.07–1.6	4	0.66	0.22–1.99	0		
	≥ 5	0			0			0			0		
	(Trend)			(0.11)			(0.12)			(0.43)			(1.0)
Liver	< 1	3	1.0	(Referent)	19	1.0	(Referent)	11	1.0	(Referent)	19	1.0	(Referent)
	1–2	4	4.1	0.89–18.8	1	0.61	0.08–4.7	7	2.7	1.0–6.9	1	0.61	0.08–4.7
	2–5	12	8.5	2.2–32.4	1	0.44	0.44–3.5	3	0.52	0.14–1.88	1	0.44	0.05–3.5
	≥ 5	2	1.4	0.21–9.2	0			0			0		
	(Trend)			(0.18)			(0.38)			(0.58)			(0.38)

Obs, number of observed cases. Analyses based on Poisson regression using the lowest exposure group as the referent group, adjusting for age and time period.

a Data from Demers et al. 2006.

b Trend *p*-value.
